# Internal luminescence efficiencies in InGaP/GaAs/Ge triple-junction solar cells evaluated from photoluminescence through optical coupling between subcells

**DOI:** 10.1038/srep38297

**Published:** 2016-12-08

**Authors:** David M. Tex, Mitsuru Imaizumi, Hidefumi Akiyama, Yoshihiko Kanemitsu

**Affiliations:** 1 Institute for Chemical Research, Kyoto University, Uji, Kyoto 611-0011, Japan; 2 Japan Aerospace Exploration Agency, Tsukuba, Ibaraki 305-8505, Japan; 3 Institute for Solid State Physics, The University of Tokyo, Kashiwa, Chiba 277-8581, Japan

## Abstract

*In-situ* characterization is one of the most powerful techniques to improve material quality and device performance. Especially in view of highly efficient tandem solar cells this is an important issue for improving the cost-performance ratio. Optical techniques are suitable characterization methods, since they are non-destructing and contactless. In this work, we measured the power dependence of photoluminescence (PL) from the InGaP and GaAs subcells of an industry-standard triple-junction solar cell. High luminescence yields enhance the luminescence coupling, which was directly verified by time-resolved PL measurements. We present a new method to determine the internal luminescence efficiencies of InGaP and GaAs subcells with the aid of luminescence coupling. High luminescence efficiencies of 90% for GaAs and more than 20% for InGaP were found, which suggest that the material quality of the grown GaAs layer is excellent while the intrinsic luminescence limit of InGaP is still not reached even for high excitation conditions. The PL method is useful for probing the intrinsic material properties of the subcells in flat band condition, without influence of transport. Since no calibration of absolute PL is required, a fast screening of the material quality is possible, which should be extremely helpful for the solar cell industry.

The spectral-splitting approach in tandem solar cells allows to extend the energy conversion efficiency of triple-junction solar cells to about twice that of a single junction solar cell^1^,^2^,^3^. It is important that the ideal Shockley-Queisser limit can be only achieved if luminescence efficiencies are unity^4^,^5^. The exceptionally high luminescence yields in high quality III-V materials can be used to enhance the interaction of the subcells in the tandem solar cell, and further push the solar cell to its limits. Having various techniques for evaluating the luminescence efficiency of a device is important for performance improvement.

The knowledge of the luminescence efficiency is essential for tandem solar cells, since the optimal bandgap combination depends on it^6^. The effect of photon recycling becomes important in materials which have both high luminescence efficiencies and high absorption coefficients, such as GaAs and metal halide perovskites^7^. It is known that high internal luminescence efficiencies can also significantly contribute to the subcell’s external quantum efficiency (EQE)^8^, due to photon recycling in a neighboring subcell, *i.e.*, photonic coupling. Since external luminescence efficiencies depend on sample geometry, the internal luminescence efficiency is more useful when characterizing a solar cell for possible performance improvement^9^. They are usually determined using electroluminescence (EL) techniques^10^,^11^,^12^,^13^,^14^. Since it is difficult to find the suited conditions for measurement, a simpler optical measurement would be favourable.

Using time-resolved photoluminescence (PL), the carrier dynamics in the subcells can be directly monitored^15^,^16^. It has been shown that solar cell properties evaluated from the PL decay curves correlate well with electrical measurements obtained under 1 sun condition^16^. By comparing the PL lifetimes obtained from a subcell under excitation of different subcells, the photonic coupling can be confirmed and the PL intensities should be related to the coupling strength convoluted with the internal luminescence efficiencies.

In this work, we present a convenient optical technique to obtain the internal luminescence efficiencies of subcells in a tandem solar cell. We measure the power dependence of the PL from the InGaP and GaAs subcells of an industry standard triple-junction solar cell. The influence of luminescence coupling was directly verified using time-resolved PL measurements. We found that the internal luminescence efficiencies can reach up to 90% for GaAs and more than 20% for InGaP, which are reasonable high values and indicate that there are no material related problems in the grown GaAs layer. The data suggests that also the InGaP material quality is high, but the material-limited internal luminescence efficiency of the InGaP layer cannot be reached even for the presently used high excitation conditions. The PL method is useful for probing the intrinsic material properties of the subcells in flat band condition, without any influence of transport. Since no calibration of absolute PL is required, a fast screening of the material quality is possible, which is important for advancing the solar cell engineering and application.

## Results

### Time-resolved photoluminescence

**Figure 1:**
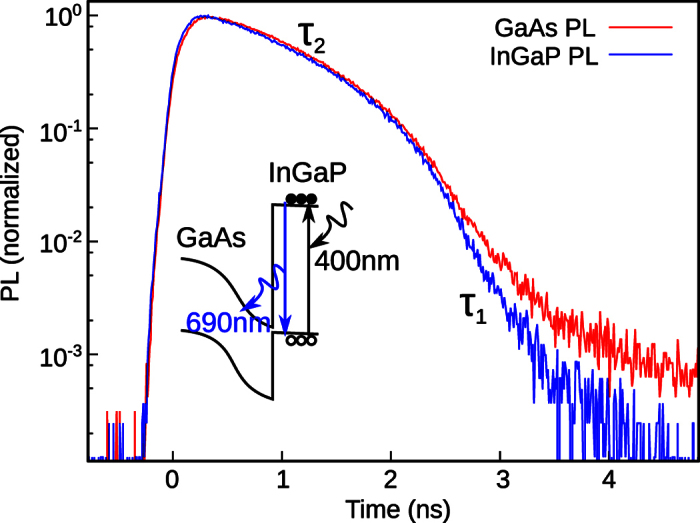
Time resolved PL of InGaP and GaAs for excitation of InGaP at 400 nm. Intensities are normalized by peak intensity. Inset: Schematic of a directly excited InGaP subcell, which is in flat-band condition and emits a part of its luminescence to the GaAs subcell which is close to short-circuit condition.

The studied triple-junction solar cell consists of a bottom Ge junction, middle GaAs junction and top InGaP junction (see Methods for details). We consider the luminescence coupling from the InGaP to the GaAs subcell, as shown in the inset of Fig. 1. In this case, the InGaP subcell is excited directly with a laser at 400 nm, and these photons are completely absorbed in the layer, as was confirmed by EQE^16^. Luminescence coupling occurs when photons emitted from the InGaP bandgap are partly transmitted and absorbed in the GaAs subcell. Although the GaAs subcell is not intentionally excited, additional carriers are created in the GaAs subcell by indirect excitation via InGaP, and these will produce a small amount of GaAs PL.

This process was confirmed with time-resolved PL (see Section Methods), and the normalized PL decays of the InGaP (blue) and GaAs (red) subcell for excitation at 400 nm are shown in Fig. 1. The analysis of the power-dependence of time-resolved PL has been proposed for characterizing electrical performance of pn-junction devices^15^. However, it can be also used to probe the coupling efficiency as explained below. Using PL decays has the advantage that no electrical contacts are needed, nor any light biasing has to be applied. The InGaP PL and GaAs PL were measured for same excitation conditions, only the short and longpass filters for detection were exchanged. A characteristic decay profile with slow component *τ*_2_ and fast component *τ*_1_ is observed. Both PL decays are very similar, with a slightly delayed GaAs PL.

After the strong pulsed excitation the carrier densities are high, and the InGaP subcell goes into flatband condition, where the effect of the electric field is minor and thus luminescence yields are high. The slow decay *τ*_2_ after the strong excitation pulse corresponds to the internal recombination under flatband condition. At about 2 ns a sudden change in the decay dynamics occurs; a faster PL decay with time constant *τ*_1_ is observed. Such a behavior is characteristic for pn-junctions, where PL is governed by charge separation^16^,^17^. We consider that at 2 ns the electric field becomes dominant again, and the charge separation can be observed^16^. It has been shown that the analysis of the slow and fast time constants can be used to analyze the electrical performance of the subcell, which should be very useful for a fast characterization of subcell performances^15^,^16^.

The GaAs PL decay follows tightly that of InGaP, with a slightly delayed onset and a longer tail. The slow decay until 2 ns is unusual for GaAs, since for the low photon flux from the InGaP subcell, a much faster decay of 0.3 ns has been confirmed for direct excitation^15^. For low PL intensities (t = 3 ns), the InGaP decay is faster than that of GaAs, since the charge separation occurs faster in the thin InGaP subcell. We consider that in this region luminescence coupling becomes much weaker, because the carrier densities are lowered and thus also the junction voltage. High luminescence coupling can improve the voltages of the subcells during solar cell operation. The onset of strong luminescence coupling is analyzed below, since the power dependence of the luminescence efficiency determines its significance for EQE.

### Power dependence of photoluminescence

**Figure 2:**
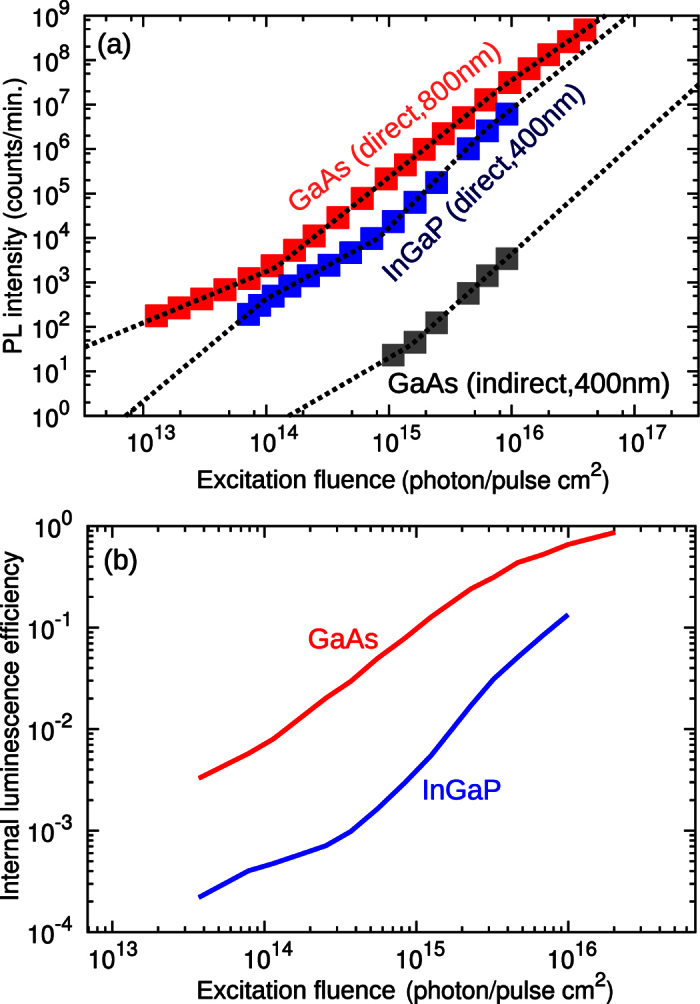
Power dependence of PL and luminescence efficiency. (**a**) Power dependence of PL intensities measured at 680 (InGaP PL) and 880 nm (GaAs PL). For InGaP (blue data), PL is for direct excitation with 400 nm. For GaAs, two data sets are shown for direct excitation with 800 nm (red data) and indirect excitation with 400 nm (gray data). (**b**) Power dependence of internal luminescence efficiency for GaAs (red) and InGaP (blue).

The power dependence of the PL intensities of the InGaP and GaAs subcells under direct and indirect excitation conditions are shown in Fig. 2(a). The InGaP PL is for excitation at 400 nm. A change in the power dependence from 1.55 to 2.8 at around 10^15^ photon/pulse cm^−2^ is observed. The PL intensity is proportional to the product of electron and hole densities. In the ideal case we expect a power dependence of 1 under low excitation powers, which corresponds to the recombination of light-induced minority carriers with the doped carriers. For high excitation powers the doped carriers can be neglected compared to the light-induced carriers and the recombination of the light-induced carriers lead to a power dependence of 2. The 50% larger values indicate that there are many nonradiative processes. This leads to an additional power dependent factor in luminescence and power dependence above the ideal one.

The PL power dependence of the GaAs subcell in our device has two data sets due to different excitation conditions. The red data set with high intensities is that obtained under direct excitation of GaAs with 800 nm. A change in the power dependence from 1.1 to 2.2 is observed at 10^14^ photon/pulse cm^−2^. This indicates that the GaAs subcell is of good quality with low carrier loss, which enables transition of the pn-junction into flat band condition already at low carrier densities. The region for power dependence near unity would correspond to the low injection region, and the region for quadratic power dependence to the high-injection condition. The gray data set with low intensities is that obtained for indirect excitation at 400 nm. It has a power dependence of 2.5, which is close to that of the InGaP subcell PL for the same excitation powers, *i.e.*, a result of luminescence coupling.

### Internal luminescence efficiencies

Now we evaluate the absolute values of the internal luminescence efficiencies, which are shown in Fig. 2(b) and explained later. This procedure usually requires measurement of absolute PL intensities, however, we found that owing to the luminescence coupling we can easily recalculate the luminescence efficiencies from the relation of the three curves in Fig. 2(a) and the input photon flux. The basic idea is that the InGaP PL intensity for direct excitation is a function of the InGaP luminescence efficiency, *f(η*_InGaP_), and the GaAs PL intensity for indirect excitation via InGaP depends on both luminescence efficiencies, *f(η*_*GaAS*_, *η*_InGaP_). Therefore, by calculating the ratio of the direct and indirect PL intensities, *η*_InGaP_ cancels out and the absolute *η*_GaAs_ is obtained. This calculation requires a knowledge of the transmission and absorption coefficients of the different layers.

For the theoretical calculation of the transmission and absorption coefficients we consider a standard five layer structure for triple-junction solar cells and define 0: Air, 1: AR_1_, 2: AR_2_, 3: InGaP, 4: GaAs and 5: Ge with their usual layer thicknesses^18^,^19^. Refractive indices were obtained from the literature^20^,^21^,^22^. The assumed anti-reflection coating consists of 2 layers, providing a suitable refractive index gradient (1.4 to 2.3)^23^, which reproduce the actual reflection spectra well^13^.

**Figure 3:**
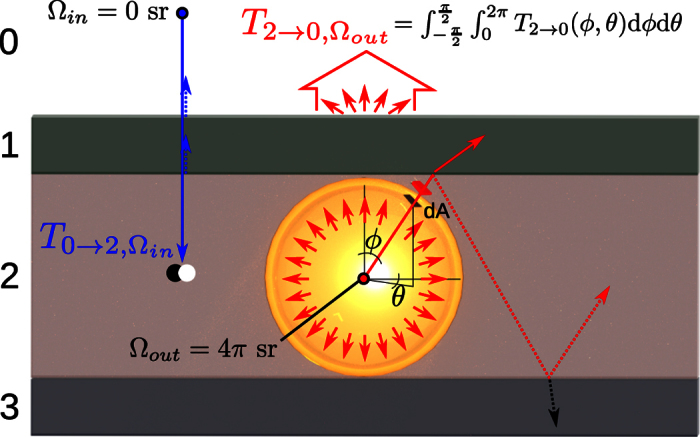
Definition of transmission coefficients for excitation and luminescence for a 4 layer structure. The incoming excitation beam (blue arrow) has no divergence (Ω_*in*_ = 0 sr). After partial reflection at the interfaces, the excitation creates electron-hole pairs due to absorption in layer 2 with intensity proportional to 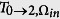. Upon recombination of the electron-hole pair, PL is emitted into all directions (red arrows, Ω_*out*_ = 4*π* sr). The sum of the transmitted rays into layer 0 will result into the total transmitted intensity 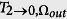.

The definitions of the transmission coefficients for excitation and luminescence are given in Fig. 3. A more general description of the approach used in this work has been already discussed in the literature^24^. The figure depicts a generalized four layer structure with successive larger refractive index and smaller bandgap for deeper layers. The blue arrow shows the excitation of layer 2 via an incoming laser beam (solid angle Ω_*in*_ = 0 sr) originating in layer 0 (air) with normalized intensity 1. After partial reflection at the interfaces of all layers, the bulk material of layer 2 absorbs the blue photons and carriers are generated according to the transmission intensity 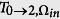. The transmission coefficient 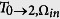 also accounts for incomplete absorption in layer 2 due to small thickness.

The luminescence process considers an emission with solid angle Ω_*out*_ = 4*π* sr. Total normalized emission intensity is 1 and each area element d*A* defined with angles *ϕ* and *θ* has equal normal photon flux. The resulting total outgoing flux 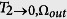 after partial reflection at the interfaces of all layers including absorption is obtained by numerically integrating over all emission angles. The cyclic reemission and reabsorption of absorbed photons is implemented later analytically.

The effective absorption coefficient 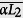 in this emission process also considers the emission into all directions. The number of absorbed photons in layer 2 must be the difference between the emission intensity (normalized to unity in our case) and the outgoing photons fluxes given with


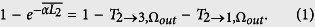


Since in layer 1 cannot absorb photons with band gap energy of layer 2, we replace 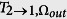 with 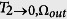. The obtained value of 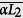 contains therefore all effects of multiple internal reflection, absorption and emission to air and substrate sides. This value cannot be obtained experimentally, since we are not able to obtain EL signals from the bottom of the sample due to the thick substrate.

The estimated light-intensity transmission and absorption coefficients for this structure are given in Table 1. They have been calculated using the transfer-matrix method^25^, where incident transmission 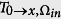 (from layer 0 Air to layer x, where x can be layer 1 through 5) and outgoing transmission 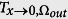 are for beam angles Ω of 0 and 4*π*, respectively. This calculation considers the 3 dimensional structure of the sample, including s and p polarization and multiple internal reflections. The total PL emission at 690 nm from the InGaP layer to air for direct excitation can be written as


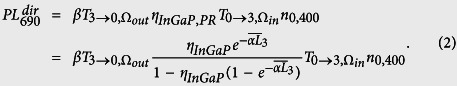


The first line in Eq. 2 is a compact notation, which explains that the InGaP PL at 690 nm for direct excitation (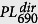) is a result of the incoming photon flux at 400 nm (*n*_0,400_), which is transmitted and absorbed in the 3rd layer (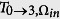), then converted into photons of 690 nm with the effective luminescence efficiency including photon recycling (PR) (*η*_InGaP,PR_), which are transmitted to air (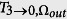) and detected with detection efficiency *β*.

The second line in Eq. 2 expands the photon recycling term *η*_InGaP,PR_ into multiple internal reflection and internal luminescence efficiency of InGaP. The effective absorption coefficient of the InGaP PL in the InGaP layer is given with 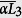, which is determined by considering the effective path length of a photon in the layer until it is absorbed again. The corresponding path length can be larger than the thickness of the layer due to emission into all 3 dimensions and total, multiple internal reflections. The fractional term comes from the assumption that the depth distribution of the re-emitted PL equals the absorption profile, and is the usual expression used to connect internal and external luminescence efficiencies^24^.

While InGaP is excited with 400 nm, the GaAs PL emission due to indirect excitation via InGaP emission at 690 nm must be considered. In analogy to the previous equation we write it as


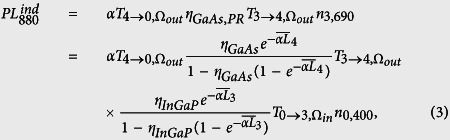


where the first line gives a short notation for easier explanation. The GaAs PL at 800 nm for indirect excitation (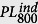) is a result of the total InGaP emission flux at 690 nm (*n*_3,690_), which is transmitted and absorbed in the 4th layer (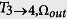), then converted into photons of 800 nm with the effective luminescence efficiency including photon recycling (*η*_GaAs*,PR*_), which are transmitted to air 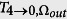 and detected with detection efficiency *α*. The ratio of both detection efficiencies 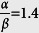 was determined with a calibrated light source. The second line expands *η*_GaAs*,PR*_ as explained above, and the InGaP emission *n*_3,690_ with the terms from Eq. 2 except the transmission to air, since now transmission to GaAs is required.

We can see that both Eqs. 2 and 3 contain *η*_*InGaP*_, which is unknown. By dividing Eqs. 2 and 3, *η*_*InGaP*_ cancels out and we obtain


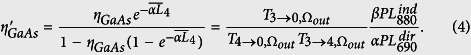


This equation contains only one unknown, *η*_*GaAs*_. The fraction of the PL intensities for direct and indirect excitation (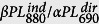) is experimentally known, and the transmission and absorption coefficients have been calculated from the layer structure. The new variable 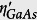 is a short notation for the photon recycling term. The corresponding GaAs luminescence efficiency *η*_*GaAs*_ can be calculated with the PL intensities for direct and indirect excitation. Because the x-value for the power dependence of indirect excitation of GaAs is the value for the power of the pulse exciting InGaP, we must consider that the actual excitation of GaAs is about 100 times smaller (x-offset of the GaAs PL data sets obtained for excitation at 400 and 800 nm). We determine *η*_*GaAs*_(3.3 × 10^13^) = 0.33% for excitation with 4.2 × 10^15^ photon/pulse cm^−2^ at 400 nm, and the total power dependence is obtained via the difference of the GaAs PL power dependence from linearity:


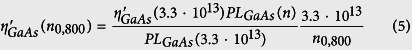


The InGaP luminescence efficiency for excitation with 4.2 × 10^15^ photon/pulse cm^−2^ at 400 nm is obtained by comparing the carrier densities in the GaAs layer for 3.3 × 10^13^ photon/pulse cm^−2^ direct excitation and 4.2 × 10^15^ photon/pulse cm^−2^ indirect excitation, which are the same since PL intensities are equal.


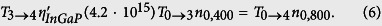


The total power dependence of InGaP is then obtained in the same manner as Eq. 5. The internal luminescence efficiency of InGaP for excitation with 3.3 × 10^13^ photon/pulse cm^−2^ at 400 nm is estimated to be about *η*_*GaAs*_(3.3 × 10^13^) = 0.02%.

The power dependence of the internal luminescence efficiencies are shown in Fig. 2(b). At low powers, the efficiencies are low, then increase quickly until they reach their maximum. The maximum is the material limited internal luminescence efficiency. *η*_*InGaP*_ reaches a maximum value of more than 20% and *η*_*GaAs*_ reaches about 90%. These values are for an excitation of 2 × 10^16^ photon/pulse cm^−2^, which sums up to *I*_*pulse*_ = 8 × 10^22^ photon/s cm^−2^ at a repetition of 4 MHz. This excitation must be compared with the carrier lifetime and the 1 sun excitation condition. The AM0 1 sun photon flux for the top cell between 350 and 690 nm is *I*_1*sun*_ = 1.5 × 10^17^ photon/s cm^2^. Since the pulsed excitation introduces a much higher carrier density for a short time, the material properties for almost perfect flat band condition can be obtained.

## Discussion

The internal luminescence efficiencies can be compared with that obtained by EL under injection current corresponding to that of one sun illumination, 59 and 6% for GaAs and InGaP, respectively^14^. The EL results are comparable to the set of efficiencies obtained by PL at excitation 5.9 × 10^15^ photon/pulse cm^−2^: 51 and 8%. The photonic coupling from the InGaP to the GaAs subcell should be therefore about 0.08*T*_3→4_ = 5%, which is in good agreement with the GaAs subcell EQE at 400 nm^13^.

Obviously, from the PL method we obtain higher maximum values than predicted by the EL method for 1 sun. During the EL measurement using a device current for 1 sun condition, the constant current through the sample restricts the working condition. In such a case it is possible that the subcell voltages are below those which build up under 1 sun illumination, and the bands are not completely flat. Therefore, the radiative rate is reduced and a lower luminescence efficiency is calculated. Another possibility might be that 1 sun illumination is insufficient to induce a flat band condition. Therefore, in any case, the EL results alone may not reflect the intrinsic material quality, since extrinsic effects arising from the series constraint are included. We consider that the high excitation densities under pulsed excitation reveal the intrinsically limited luminescence efficiencies of the subcells, since an almost perfect flat band condition can be reached. This enables to characterizing the subcells material quality in a fast and accurate way without influence of transport losses. The saturation of the curve for GaAs at high photon fluxes in Fig. 2(b) confirms that the intrinsically limited luminescence efficiency is about 90%. On the other hand, we consider that the material-limited value of the InGaP subcell should lie well above 20%, since the curve is still not saturated even at highest photon fluxes in our experiment. One possible reason for this is that the flat-band condition could not be reached due to the extreme thin InGaP layer, which induces high internal electric fields.

The present PL method provides complementary information for analyzing the subcells performance. The EL method probes the luminescence efficiencies including transport, which were reduced for the top and middle subcell of our device^13^. These EL efficiencies can be either due to poor material quality or due to the junction design. Owing to the high efficiencies obtained by PL method we can infer that the material qualities are not an issue for the present device.

The validity of the present method can be confirmed by comparing the actual open circuit voltage *V*_*oc*_ and the ideal open circuit voltage *V*_*oc,ideal*_, which are related via^4^


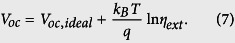


Here *k*_*B*_ is the Boltzmann constant, *T* the cell temperature, *q* the elementary charge, and *η*_ext_ the external luminescence efficiency. *V*_*oc,ideal*_ accounts for the effect of incomplete absorption, the thermalization and the solid angle of the sun illumination^26^. In case of the GaAs subcell, the *V*_*oc*_ for 1 sun was determined to be 1.01 V, which is a reliable value since the predicted device I-V is in excellent agreement with the actual I-V curves^13^. The external luminescence efficiency is calculated with *η*_ext_ = *η*_*GaAs,PR*_(T_4→0_+*T*_4→5_) = 18.5%. The upward directed photon flux sums up to 1.05%, which is similar to the value measured with EL^14^. By inserting the internal luminescence efficiencies obtained in this work into Eq. 7 we obtain 1.04 V, which is in fair agreement with the experimental value. For the InGaP subcell we obtain 1.42 V, which is slightly higher than the 1.36 V predicted for 1 sun using EL^13^.

Overall, the PL method provides values which are consistent with the electrical measurements. The 30 to 60 meV higher voltages predicted with the optically obtained luminescence efficiencies may be a result of the fact that the material limited value can be only obtained for excitation conditions which lie above 1 sun. In order to match the voltages with those obtained from EL, we need to insert lowered values of 57 and 2.6% for the GaAs and InGaP luminescence efficiencies, respectively. From Fig. 2(b) we find that these values can be obtained for pulsed photon fluxes of about 7 × 10^15^ and 3 × 10^15^ photon/pulse cm^−2^, which can be thus considered equivalent to 1 sun condition.

We note that Eq. 7 is used to relate *V*_*oc*_ of a solar cell with the EL signal of the device in light emitting diode (LED) mode^26^. In such a case one usually considers electrically injected carriers in steady state condition, and *η*_*ext*_ means the amount of injected carriers contributing to luminescence in relation to the total injected carriers. Our experiment uses optical injection with a short pulse under open-circuit condition. We determine the amount of injected carriers contributing to radiative recombination using the emission of PL, which is a transient process. In order to apply Eq. 7 we have to use PL signals which correspond to an almost steady-state band condition as in the LED working condition, *i.e.*, flat band condition. This can be achieved when we apply strong pulsed excitation. Initially the device is in short-circuit condition, but after strong excitation a flat band condition for a few tenths of nanoseconds is obtained, meaning we induce the LED operation for a few tenths of nanoseconds^15^,^16^. Theoretical analysis showed that PL from short-circuit condition can contribute to the luminescence from a junction at elevated voltage^27^,^28^,^29^. However, the PL from short-circuit condition is a minor component which occurs at later times under our strong excitation condition^16^. Therefore, the use of Eq. 7 should be a good approximation for our experiment, reflected in the reasonable values obtained for both subcells.

In summary, it was shown that the power dependence of PL obtained for direct excitation and indirect excitation via luminescence coupling allows to determine the internal luminescence efficiencies of the subcells. The GaAs subcell has very high material quality and also the InGaP subcell seems to approach a remarkable high luminescence efficiency at high excitation fluxes. Due to the pulsed excitation, the PL method is useful for probing the intrinsic material properties of the subcells in flat band condition, without any influence of transport.

The most important practical value of our PL method for other materials systems and devices is the derived Eq. 4, which allows to determine the luminescence efficiency without the difficult absolute PL measurements. This method is applicable to all systems with optical coupling between two materials. We believe that the optical characterization provides very useful fast screening technique of solar cells.

## Methods

### Sample

The studied triple-junction solar cell consists of a bottom Ge junction, middle GaAs junction and top InGaP junction^30^. This device has a conversion efficiency of about 27%. The EQE and PL spectra of the subcells have been discussed in our previous works^13^,^15^. From PL we confirmed that the InGaP bandgap energy is 1.8 eV (690 nm), and that of GaAs is 1.42 eV (875 nm). The 400-nm light is completely absorbed in the top InGaP subcell. Illumination with 800-nm light excites the middle GaAs subcell, without exciting the top InGaP subcell.

### Photoluminescence

Spectral information of PL from InGaP and GaAs was obtained by using a 50 cm monochromator and a Si charge-coupled-device. Luminescence coupling was measured with an avalanche photo diode and a Ti:Sapphire laser for excitation with a pulse width of about 200 fs at 800 nm and a repetition rate of 4 MHz. 400 nm light was obtained using a beta barium borate crystal. Excitation spot sizes were about 20 and 10 *μ*m for 800 and 400 nm excitation, respectively. The optical measurements were performed at room temperature and under open circuit condition.

## Additional Information

**How to cite this article**: Tex, D. M. *et al*. Internal luminescence efficiencies in InGaP/GaAs/Ge triple-junction solar cells evaluated from photoluminescence through optical coupling between subcells. *Sci. Rep.* **6**, 38297; doi: 10.1038/srep38297 (2016).

**Publisher's note:** Springer Nature remains neutral with regard to jurisdictional claims in published maps and institutional affiliations.

## Figures and Tables

**Table 1:** Properties of the multi-layer structure.

	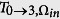	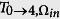	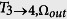	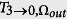	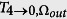	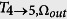	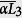	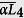
400 nm	0.982	—	—	—	—	—		
680 nm	—	—	0.595	0.022	—	—	0.48	
800 nm	—	0.988	—	—	—	—		
880 nm	—	—	—	—	0.015	0.248		1.34

The theoretically obtained light transmission coefficients *T* between air, InGaP and GaAs layers for s-polarization (results for p-polarization are almost identical), and the effective light absorption coefficients 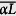 for photon recycling.
